# A VHH-Based Anti-MUC1 Chimeric Antigen Receptor for Specific Retargeting of Human Primary T Cells to MUC1-Positive Cancer Cells

**DOI:** 10.22074/cellj.2021.6917

**Published:** 2020-04-22

**Authors:** Alireza Rajabzadeh, Fatemeh Rahbarizadeh, Davoud Ahmadvand, Maryam Kabir Salmani, Amir Ali Hamidieh

**Affiliations:** 1. Department of Tissue Engineering and Applied Cell Sciences, School of Advanced Technologies in Medicine, Tehran University of Medical Sciences, Tehran, Iran; 2. Department of Medical Biotechnology, Faculty of Medical Sciences, Tarbiat Modares University, Tehran, Iran; 3. Department of Biochemistry, School of Allied Medical Sciences, Iran University of Medical Sciences, Tehran, Iran; 4. Department of Stem Cell and Regenerative Medicine, National Institute of Genetic Engineering and Biotechnology (NIGEB), Tehran, Iran; 5. Pediatric Cell Therapy Research Centre, Tehran University of Medical Sciences, Tehran, Iran

**Keywords:** Immunotherapy, Single-Chain Antibodies, T-Lymphocytes

## Abstract

**Objective:**

Immunotherapy with redirected T cells that express a chimeric antigen receptor (CAR) is a promising prospect in cancer treatment. Most CARs use murine-derived single-chain variable fragments (scFvs) as an antigen targeting moiety, which may lead to host immunogenic responses and engineered T cell disappearance. It seems that development of less immunogenic CARs, such as CARs composed of the camelid variable domain of heavy chain antibodies (VHHs) may likely overcome this obstacle. Here, we improved the expression of the VHH-based anti-MUC1 CAR gene construct using a third generation lentiviral vector in primary human T cells and assessed its effect on antigen specific targeting, activation and cytotoxicity of redirected human T cells.

**Materials and Methods:**

In this experimental study, we established a second generation novel CAR (VHH-based anti- MUC1 CAR) that contained a camelid-derived anti-MUC1 VHH followed by an IgG3 hinge, a CD28 transmembrane domain and signalling endodomains of CD28 and CD3^+^. Next, we constructed lentiviral vectors that contained this CAR gene construct using an optimized transiently virus production method and transduced it into human T cells. Cell surface expression of CAR, cytokine secretion and cytotoxic activity were assessed in the transduced CD3+T cells.

**Results:**

The transduced T cells had high levels of surface expression of CAR. T cells that expressed anti-MUC1 CAR showed significantly increased secretion of Th1 cytokines, including IL-2, TNF alpha and IFN-γ, as well as cytotoxic activity upon recognition of MUC1 on tumour cells after co-incubation with T47D or MCF-7 (MUC1-positive) compared with A431 (MUC1-negative) or untransduced T cells.

**Conclusion:**

Our results suggested that, given the unique properties of VHHs to prevent immunogenic responses and tonic signalling, our novel VHH-based anti-MUC1 CAR might be effective for clinical purposes in cancer immunotherapy.

## Introduction

Adoptive cell-based immunotherapy is a promising approach in the treatment of cancers. In recent years, chimeric antigen receptor (CAR)-T cell based immunotherapies have shown impressive successes in the treatment of hematologic malignancies, especially lymphoma. In 2017, the first two engineered cell-based immunotherapies (CD19-targeted CAR-T cells) were approved by the US Food and Drug Administration (FDA). Kymriah was introduced by Novartis for treatment of B-cell acute lymphoblastic leukaemia (ALL) patients less than 25 years of age and about two months later, Yescarta was introduced by Kite Pharma (Gilead) for patients with relapsed or refractory B-cell lymphoma (1, 2).

Despite the significant advances in CAR-T therapies against hematologic malignancies, the treatment of most solid tumours is still faced with serious challenges (3). One of the main reasons for this failure is the identification of suitable tumour-specific antigens for solid tumours. Contrary to CD19, which is expressed solely on the surface of B-lymphocytes, most tumour antigens for solid tumours are found in healthy tissues, which can be recognized by redirected T cells and lead to "on-target offtumour" toxicities. For instance, Lamers et al. (4) have observed infiltration of cytotoxic T cells that surrounded the bile duct in renal cell carcinoma patients who received transfusions of anti-CA IX CAR-T cells. They reported that the normal bile duct epithelial cells expressed CA IX.

The physical barrier of solid tumours is another challenge. In solid tumours, unlike hematologic malignancies, CAR-T cells must successfully migrate from the blood to the tumour site, pass across the tumour stroma, and identify the antigen. Unfortunately, there is often a "chemokine receptor/chemokine mismatch” between CAR-T cells and tumours as well as “poor trafficking" after adoptive CAR-T cell transfer. Another challenge is the presence of an immunosuppressive system around the tumour that blocks the effectiveness of CAR-T cells. The presence of infiltrating regulatory T cells, checkpoint pathways (such as PD-1/PD-L1), inhibitory cytokines (TGF-β and IL-10), and a hostile tumour microenvironment (hypoxia, acidic pH, oxidative stress, etc.) in most solid tumours has presented problems for CAR-T cell therapy. Currently, several novel innovations such as Identification of neoantigens, designing inhibitory CARs (iCARs), dual recognition of different antigens by two CARs (tandem CARs), co-expression of cytokine receptor transgenes in CAR-T cells, and the combination of checkpoint inhibitors with CAR-T cells have been developed to overcome these barriers (3, 5).

In addition, the long-term persistence and proliferation of infused CAR-T cells is a determining factor that dominates the inefficiency observed in CAR-T treatments for solid tumours. Recently, several studies have reported poor persistence of engineered T cells after adoptive infusion due to immunogenicity of the *CAR *transgene (6-8). Development of a host immune response against mouse derived single-chain variable fragments (scFvs) is one of the main reasons for depletion of CAR-T cells after infusion (9). A strategy to reduce CAR immunogenicity and subsequently improve therapeutic efficacy in CAR-T cell therapies would be the use of humanized mouse derived scFvs (10, 11) or totally human scFvs (12-14) in the CAR structure. Unfortunately, the use of humanized scFv does not prevent the development of anti-IgE responses that induce anaphylaxis (8). Furthermore, in a clinical trial, despite the use of humanized scFv TAG-72-binding domain in the CAR construct, anti-CAR immunogenic responses resulted in rapid clearance of infused CART72 cells in most patients (15). An alternative strategy to overcome this obstacle is the employment of camelid variable domain of heavy chain antibodies (VHH) instead of scFv in the CAR anatomy (16, 17). These antibodies, also known as Nanobody®, present an extensive antigen binding repertoire and high binding affinity, despite the lack of a light chain.

In addition to naturally transmitting the stimulatory signals, CARs may constitutively trigger antigenindependent tonic signalling. These frequent signals can lead to T cell exhaustion and negatively affect antitumor efficacy *in vivo*. Antigen-independent tonic signalling, at least in part, is due to self-aggregation characteristics of the scFvs used in CARs. Various strategies have been suggested to reduce antigen-independent signalling, including targeting of CARs to the endogenous TCR alpha (*TRAC*) gene locus, utilization of self-inactivating (SIN) lentiviral vectors instead of gamma-retroviruses, incorporation of 4-1BB in endodomain signalling of CARs and substitution of single-domain antibodies instead of scFv (18). Therefore, the use of VHHs in the CAR structure seems to enhance the functional efficacy of redirected T cells *in vivo*.

Our main objective in designing this study was the stable expression of the VHH-based anti-MUC1 *CAR* gene construct, which we previously constructed in primary human T cells using a third generation lentiviral vector. We also sought to investigate the efficiency of this chimeric receptor on the activation and cytotoxicity of redirected human T cells. Accordingly, we constructed lentiviral particles that contained second-generation VHH-based anti-MUC1 CAR. Subsequently, we described the *in vitro* functional activity of VHH-based anti-MUC1 CAR-T cells by cytolysis of MUC1-positive tumour cells and cytokine production.

## Materials and Methods

### Cell lines, antibodies and reagents

In this experimental study, human breast cancer cell lines (T47D and MCF-7 cells), human epidermoid squamous carcinoma cell line (A431), and Lenti-X 293T cells were purchased from Iranian Biological Resource Centre (IRBC, Iran). T47D cells were cultured in RPMI 1640 medium (Gibco, Life Technologies, USA). MCF-7, A431 and Lenti-X 293T cells were cultured in Dulbecco’s Modified Eagle Medium (DMEM, Gibco, Life Technologies, USA). Both media were supplemented with 10% fetal bovine serum (FBS) and 2 mM L-glutamine (Gibco, Life Technologies, USA). Furthermore, for packaging and production of lentiviral particles, Lenti-X 293T cells were maintained in DMEM (Gibco, Life Technologies, USA) that consisted of 10% FBS and 2 mM L-glutamine. For flow cytometry assessment, APCconjugated anti-CD3 (BD Pharmingen^TM^) was purchased from BD Biosciences (USA) and FITC-conjugated goat anti-rabbit IgG secondary antibody was purchased from Abcam (Cambridge, MA, USA).

### Vectors and preparation of anti-MUCI chimeric antigen receptor gene construct

pLJM1-EGFP (Addgene plasmid #19319) transfer plasmid (a gift from David Sabatini), pRSV-Rev), pMDLg/ pRRE and pMD2.G plasmids were used to produce the third generation lentiviral particles. Anti-MUC1 CAR cassette that contained coding sequences for anti-MUCI VHH-IGg3-CD28-CD3^+^ was made in our previous study (19). This construct was modified and PCR amplified by oligonucleotide primers

F: 5ˊ-TATAGCTAGCGCCACCATGGCCGAGGTGGAG- 3ˊ and

R: 5ˊ-TATTACCGGTTTCGATCCTCCTCC-3ˊ

designed to introduce the NheI (3ˊ site) and AgeI (5ˊ site) restriction sites in the ends of the CAR construct. The modified anti-MUCI construct was subcloned to the pLJM1-EGFP plasmid using digestion and ligation of the NheI/AgeI fragment.

### Transfection and viral packaging

First, to determine the optimum transfection condition, 0.8×10^6^ Lenti-X 293T cells were seeded onto a 24-well plate and incubated overnight in a 37˚C, 5% CO_2_ incubator. The next day, pMDLg/pRRE, pRSV-Rev, pMD2.G and pLJM1-EGFP plasmids were co-transfected to the Lenti-X 293T cells using branched polyethyleneimine (PEI, Sigma-Aldrich, DE) (20) in PEI:DNA ratios of 1:1, 2:1 and 3:1; incubation times of 3, 6 and 24 hours; and various PEI concentrations (0.25 μg/μL, 0.5 μg/μL, 0.75 μg/μL and 1 μg/μL). After 24 hours, we evaluated the transfection rate by (GFP) expression using a fluorescent microscope. Cell viability was appraised by cell counting using the dye exclusion test (trypan blue). For preparation of recombinant viral particles, 2.5×10^6^ Lenti-X 293T were cultured on 100 cm^2^ plates and incubated overnight in a 37˚C, 5% CO_2_ incubator. Afterwards, the pLJM1-CAR recombinant vector along with helper vectors (pRSVRev, pMDLg/pRRE, pMD2.G) were co-transfected to the Lenti-X 293T cells by the optimized PEI transfection method. Cell supernatants that contained the virus particles were harvested at 24, 48 and 72 hours after transfection and were centrifuged at 500× g/4˚C. The supernatants that contained the virus particles were filtered through 0.45 μm filters (Millipore) to remove cell debris, then transferred to sterile capped tubes and concentrated by ultra-centrifugation (20000 × g for 2 hours at 4˚C). The probable virus pellet was resuspended in 100-200 μL of DMEM, from which we obtained aliquots that were stored at -80˚C until the subsequent transduction step.

The infective lentivirus titre was determined by quantitative PCR (21) for the puromycin resistance gene using the following

pur-F: 5ˊ-GCAGCAACAGATGGAAGG-3ˊ and

pur-R: 5ˊ-GAGGTCTCCAGGAAGGC-3ˊ primers.

Briefly, after transduction of Lenti-X293T cells, copy numbers of integrated lentiviral vectors were measured by the standard curve of real-time PCR (Rotor-Gene 6000 Series Software 1.7) and the following formula: (DNA amount (ng)×6.022×10^23^)/(length (bp)×1×10^9^×650). The titre of the recombinant anti-MUC1 CAR lentiviral vector, reported in transforming units (TU)/mL, was calculated to be 1.12×10^8^ TU/mL.

### Human T cell culture and transduction

Peripheral blood samples were obtained from healthy males and non-pregnant females between the ages of 25-35 years in compliance with the Institutional Review Board.approved research protocols of the Tehran University of Medical Sciences (IR.TUMS. VCR.REC.1395.538). All blood samples collected following donor informed consent. Peripheral blood mononuclear cells (PBMC) were isolated by the Ficollpaque (Sigma, GE) density gradient separation method. Freshly isolated PBMCs were cultured in 2 mL 10% FBS RPMI1640 media supplemented with 100 IU/ mL rIL-2 (Miltenyi Biotech, DE) and subsequently mixed with Dynabeads Human T-Activator CD3/ CD28 (Gibco by Life Technologies, USA) at a 1:1 (bead:cell) ratio. At 48 to 72 hours after activation, the T cells were transduced with recombinant lentiviral particles at a multiplicity of infection (MOI) of >20 using the spinoculation protocol where the MOI was calculated by viral titre/number of cells. Briefly, 2×10^5^ T cells were resuspended in 1 mL of complete media (RPMI 1640, 5% FBS, 2 mM L-glutamine) to which we added the appropriate volume of concentrated virus particles and soluble RetroNectin® (Clontech) for a final concentration of 8 μg/mL. Cell suspensions were centrifuged at 800 × g for 90 minutes at 32˚C. T cells were then resuspended in complete media supplemented with 100 IU/mL rIL-2 and plated in 24-well plates for 72-96 hours (humidified 5% CO_2_, 37˚C incubation).

### Flow cytometry

In order to detect VHH-based CAR expression on T cells, FITC goat anti-rabbit IgG antibody, specific for VHH (Abcam, Cambridge, UK), was used to stain 2×10^5^ transduced T cells. The purity of the activated T cells was verified by APC mouse anti-human (BD Pharmingen^TM^, CA). All samples were examined by BD FACSCanto II equipment. FlowJo software (v10) was utilized for data analysis.

### Analysis of cytokine production after co-cultivation of transduced T cells with tumour cells

To examine antigen specific activation and cytotoxicity of the transduced T cells, confluent cells that highly expressed MUC1 (T47D and MCF-7) and also cells with no or slight expression (A431) were co-cultured with transduced T cells at a 10:1 (effector:target) ratio in RPMI 1640 (10% FBS) supplemented with rIL-2 (100 IU/mL) (n=3). The untransduced T cells were co-cultured with cancerous cells and a single culture of tumour cells were used as the negative control in this assay. After 72 hours of incubation, all supernatants were collected and cytokine secretion was measured by the human TNF alpha (Abcam, USA), human IL-2 (Abcam, USA) and IFN-γ (Abcam, USA) ELISA assays according to manufacturer’s instructions.

### Cell viability assay for tumour cells after co-culturing with anti-MUCI chimeric antigen receptor T cells

Viable MUC1-positive or MUC1-negative cells were analysed after co-culture with either anti-MUCI CAR-T cells or untransduced T cells in a 10:1 ratio by the MTT assay (n=3). For this purpose, after 72 hours, the anti- MUC1 CAR-T cells or untransduced T cells were removed and MTT reagent was added directly into the cell media. Subsequently, the cells were maintained for 3 hours in a humidified 5% CO_2_ incubator at 37˚C. Cell supernatant media were aspirated after the incubation time and the formation of a formazan product (dissolved in DMSO) was detected at a 540 nm absorbance wavelength by an ELISA plate reader.

Analysis of variance (ANOVA) was used to identify differences between groups. Data were analysed by ANOVA and Tukey’s post hoc test using GraphPad Prism software (version 7.05). The data are shown as mean ± SEM or mean ± SD. P<0.05 was considered statistically significant.

## Results

### Preparation of variable domain of heavy chain antibodies-based anti-MUC1 chimeric antigen receptor construct

**Fig.1:**
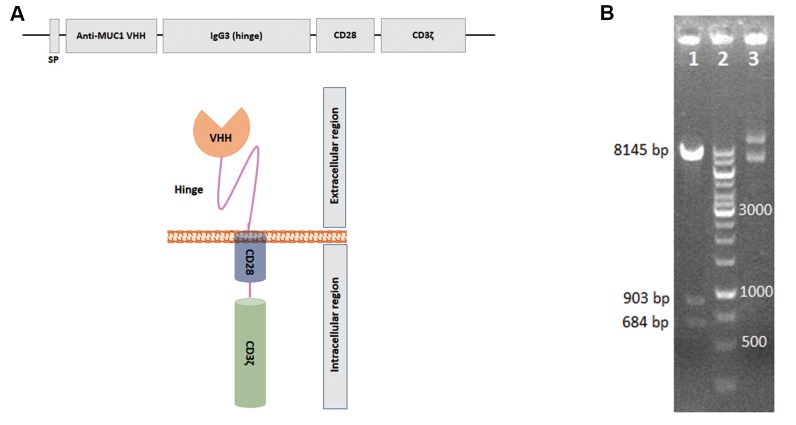
Design and sub-cloning confirmation of variable domain of heavy chain antibodies (VHH)-based anti-MUC1 chimeric antigen receptor (CAR). **A. **Schematic representation of VHH-based anti-MUC1 CAR that contains signal peptide (SP), the anti-MUC1 VHH, IgG3 hinge, and CD28 and CD3و, as intracellular domains. **B.** Insertion of VHH-based anti-MUC1 *CAR* gene construct into the pLJM1 vector was confirmed by the enzymatic digestion test. The recombinant vector digested by BsrG1 restriction enzyme produced 684 base pair (bp), 903 bp and 8145 bp fragments (line 1). Line 2; DNA ladder and Line 3; Undigested pLJM1-anti-MUC1 CAR plasmid.

The VHH-based anti-MUC1 CAR construct has been generated previously (19). This gene construct is comprised of a sequence coding camelid VHH antibody, an IgG3 domain (hinge), the transmembrane sequence of human CD28 and signalling enodomains that include CD28 and CD3^+^. The anti-MUCI VHH-IgG3 hing-CD28- CD3^+^ construct was successfully cloned into the pLJM1- EGFP plasmid. Sub-cloning was verified by restriction enzyme digestion and DNA sequencing according to the Sanger sequencing method (16). The recombinant vector was determined to be 9732 bp that resulted in fragments of 648 bp, 903 bp and 8145 bp when digested by the BsrG1 restriction enzyme. Undigested recombinant plasmids were observed in a supercoil form (Fig.1).

### Transfection optimization, production of recombinant lentivirus particles that contained variable domain of heavy chain antibodies-based anti-MUC1 chimeric antigen receptor

The optimal PEI:DNA ratio, transfection incubation time and PEI concentration were identified to limit PEI toxicity side effects as well as to maximize transfection efficacy.

**Fig.2:**
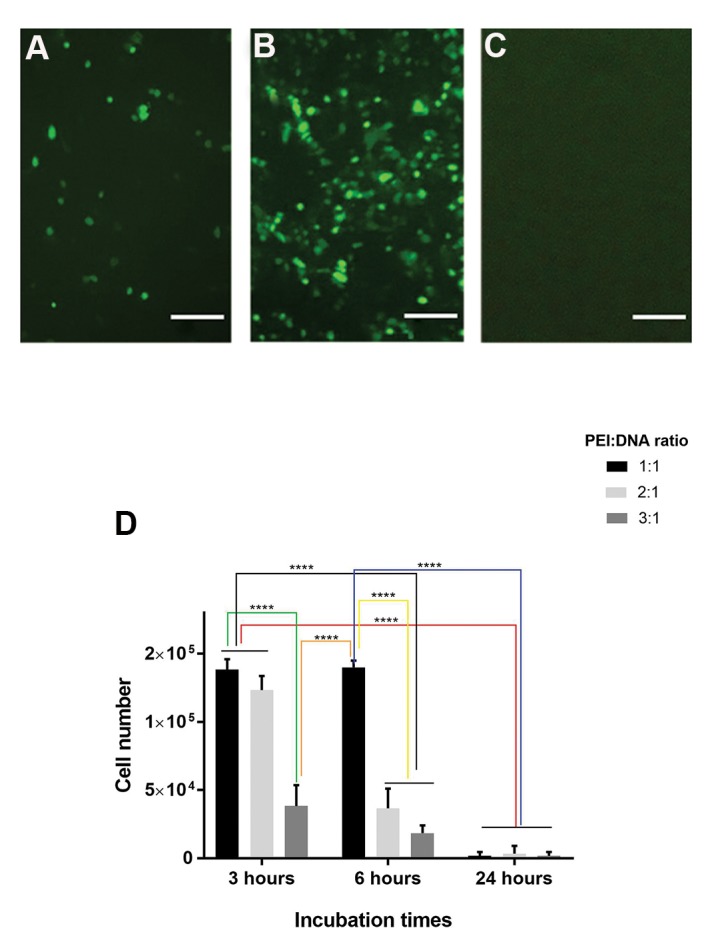
Optimization of the polyethyleneimine (PEI):DNA ratio and incubation time for optimal transfection of Lenti-X 293T cells. **A-C.** The transfection efficiency of pLJM1-EGFP vector (empty backbone) was verified based on fluorescence microscopy of green fluorescence protein (GFP) expression. The upper images show the effectiveness of vector transfection at **A.** 3, **B.** 6 and **C.** 24 hour incubation times of PEI-DNA complexes (1:1 ratio). **B.** The maximum levels of GFP fluorescence was observed at the 6 hour incubation time (scale bars: 50 μm). **D.** The graph indicates number of viable cells after PEI-based transfection at various incubation times and PEI:DNA ratios. The results show that the 24 hour incubation time of PEI or PEI:DNA 3:1 ratio are cytotoxic. Error bars represent the SD of 3 samples. ****; P<0.001 was considered statistically significant.

**Fig.3:**
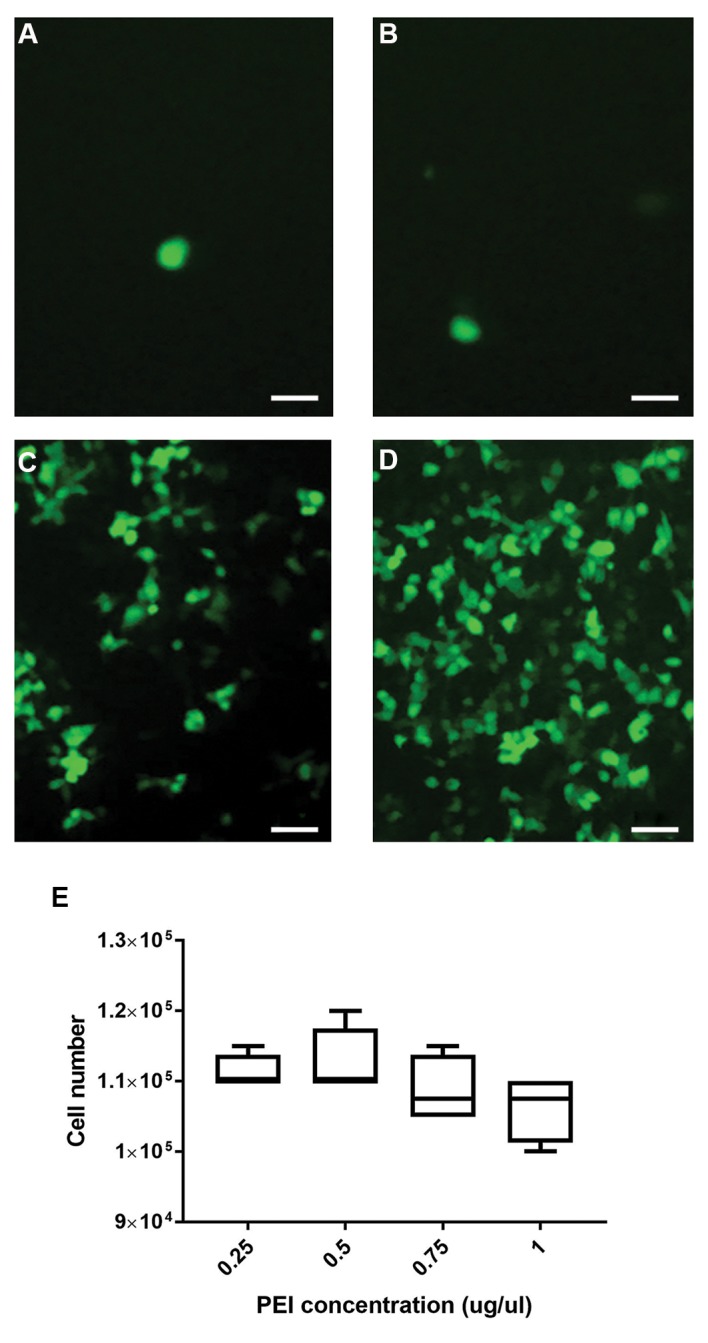
The effect of various concentration of polyethyleneimine (PEI) on transfection efficiency and viability of Lenti-X 293T cells in PEI:DNA at a 1:1 ratio and 6 hour incubation time. The effect of **A.** 0.25 μg/μl, **B.** 0.5 μg/μl, **C.** 0.75 μg/μl and 1 μg/μl of PEI concentrations on transfection on pLJM1-EGFP (empty backbone) as shown by green fluorescence protein (GFP) expression. **D.** The images indicate the highest levels of GFP expression at the 1 μg/μl PEI concentration (scale bars: 50 μm). **E.** The graph indicates the numbers of viable cells after transfection of the pLJM1-EGFP vector into Lenti-X 293T cells using various concentrations of PEI. Error bars represent the SD of 4 samples.

As shown in Figure 2, 24 hours incubation of the PEI-DNA complex, regardless of the PEI:DNA ratio, led to the death of the Lenti-X 293T cells and no green fluorescence expression. In the 3 hour incubation time, both the 1:1 and 2:1 PEI:DNA ratios resulted in low levels of fluorescence (Fig.2A), while the 3:1 PEI:DNA ratio was cytotoxic (Fig.2D). The 6 hour incubation of Lenti-X 293T cells with the PEI-DNA complex showed the highest level of fluorescence at the 1:1 PEI:DNA ratio (Fig.2B), while the PEI:DNA ratios above 1:1 caused cell death (Fig.2D). Figure 3 shows the viability profiles and transfection efficiency of the transfectants in various PEI concentrations where the condition of transfection was considered the 1:1 PEI:DNA ratio at the 6 hour incubation time. Cell viability density in cells treated with PEI concentrations (μg/μL) of 0.25, 0.5, 0.75 and 1 did not show any significant difference (Fig.3E), while the highest level of GFP expression was shown at the 1 μg/μL PEI concentration (Fig.3A-D). Hence, the 1:1 PEI:DNA ratio, incubation time of 6 hours and PEI concentration of 1 μg/μL were selected for next step of transient virus production. Transfection efficacy for the recombinant lentiviral vector was considered based on transfection of the empty vector that contained GFP as the positive control and by using the optimized protocol. The transfection rate of the GFP-vector was >80% and was calculated by using a fluorescent microscope and counting the cells that emitted fluorescence.

### Transduction of recombinant lentiviral particles into human primary CD3+ T cells

**Fig.4:**
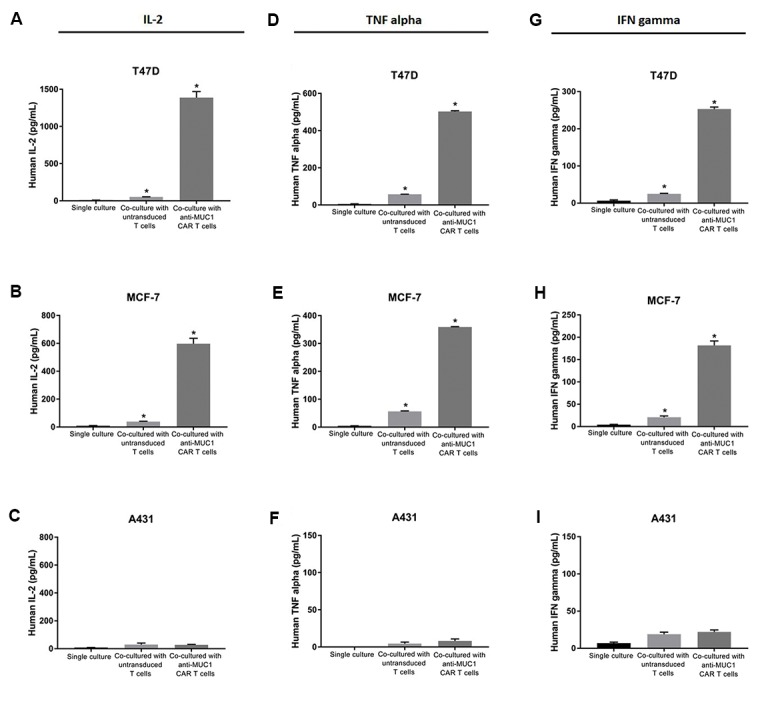
Immunophenotyping of activated CD3^+^ T cells for detection of chimeric antigen receptor (CAR) expression after transduction of anti-MUC1 CAR recombinant lentiviruses. **A.** Flow cytometry was utilized to characterize peripheral blood mononuclear cells (PBMCs) for the T cell surface marker (CD3) after 72 hours of activation with CD3/CD28 dynabeads. Cells stained with APC-conjugated anti-CD3 antibody. **B.** Indicates unstained flow cytometry of transduced T cells. **C.** Represents CAR expression on CD3^+^ T cells after APC-conjugated anti-CD3 and FITC-conjugated anti-VHH staining.

After stimulation of PBMCs, flow cytometry data revealed positive detection of the CD3 marker on the stimulated PBMCs. The results showed that the cell population of CD3^+^ T cells was almost 90%. (Fig.4A).

Flow cytometry results of transduced T cells showed a high percentage of simultaneous expression of anti-MUC1 CAR and CD3 surface marker on these cells (84.9%), which were called anti-MUC1 CAR-T cells (Fig.4B, C).

### Cytokine secretion of anti-MUC1 chimeric antigen receptor-T cells

**Fig.5:**
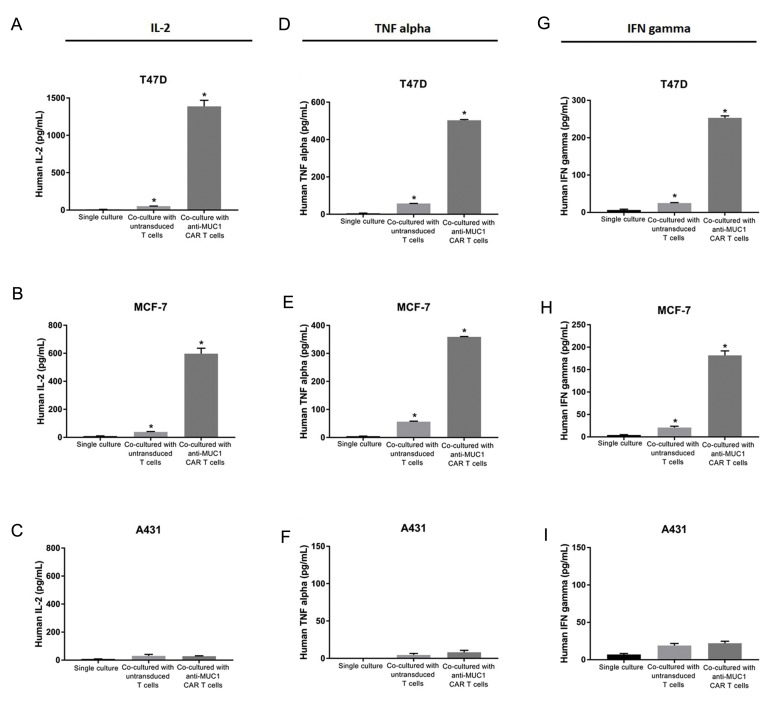
Cytokine production of anti-MUC1 chimeric antigen receptor (CAR)-T cells. Anti-MUC1 CAR-T cells or untransduced T cells co-cultured for 72 hours with MUC1-positive cells (T47D and MCF-7) or MUC1-negative cells (A431). **A-C.** Concentrations of IL-2, **D-F.** TNF alpha and **G-I.** IFN-γ secreted into supernatants was quantified by the enzyme-linked immunosorbent assay (ELISA). A single culture from each of cell lines was maintained as the negative control. The results are shown as the mean ± SD of three samples (n=3). *; P=0.033.

Cytokine production is a functional hallmark of CAR-T cells. Therefore, production of cytokines IL-2, TNF alpha and IFN-γ were measured using ELISA after co-culturing the modified T cells with MUC1 positive (T47D and MCF-7) or MUC1 negative (A431) tumour cell lines. The results revealed that the MUC1-redirected CAR-T cells could produce TNF alpha, IFN-γ and IL-2 in response to recognition of MUC1 expressed on the tumour cells (Fig.5). The co-culture of T47D tumour cells significantly triggered the production of IL-2 (1388 ± 81.32 pg/mL, 28-fold, Fig.5A), TNF alpha (503 ± 4.24 pg/mL, 9-fold, Fig.5D) and IFN-γ (253 ± 5.65 pg/ mL, 10-fold, Fig.5G) from MUC1-specific CAR-T cells, but not the untransduced T cells. Furthermore, MUC1- specific CAR-T cells significantly increased secretion of IL-2 (597.5 ± 38.89 pg/mL, 15-fold, Fig.5B), TNF alpha (359 ± 1.41 pg/mL, 6-fold, Fig.5E) and IFN-γ (182 ± 9.89 pg/mL, 9-fold, Fig.5H) in response to MCF-7, another MUC1 positive tumour cell line. The untransduced T cells did not show significant production of IL-2 (Fig.5B), TNF alpha (Fig.5E) or IFN-γ (Fig.5H) after the co-culture with MCF-7 cells. When MUC1-negative tumour cells (A431) were used as the stimulator, MUC1-specific CAR-T cells showed no significant secretion of IL-2 (30 ± 2.82 pg/mL, 1-fold, Fig.5C), TNF alpha (8 ± 2.82 pg/mL, 1.7-fold, Fig.5F) and IFN-γ (22 ± 2.82 pg/mL, 1.2-fold, Fig.5I). A single culture from each of cell lines was maintained as the negative control.

### Cytotoxicity of MUC1-redirected chimeric antigen receptor-T cells

**Fig.6:**
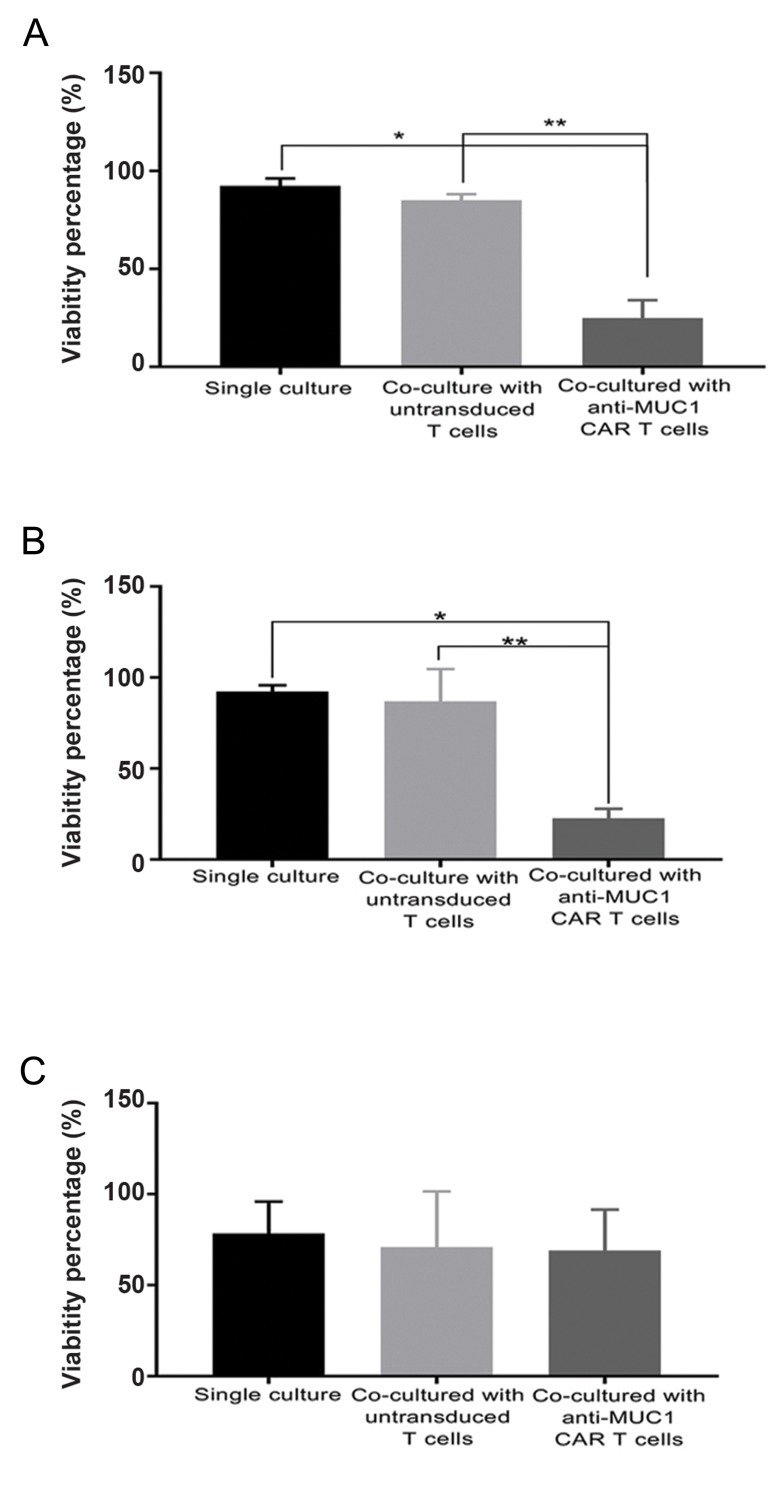
Cytotoxicity mediated by anti-MUC1 chimeric antigen receptor (CAR)-T cells. Viability of MUC1-positve cells [**A.** T47D and **B.** MCF-7) or MUC1-negative cells (**C.** A431) measured by the MTT assay after 72 hours co-incubation with anti-MUC1 CAR-T cells or untransduced T cells. A single culture from each of cell lines was maintained as a negative control. The results are shown as the mean ± SD of three samples (n=3). ). *; P=0.033 and **; P=0.002.

We sought to appraise the cytotoxicity of CAR-T cells by assessing cell viability of the target cells after a 72-hour co-culture with transduced or untransduced T cells (Fig.6). MTT assay results indicated that coculturing of T47D (a MUC1-positive tumour cell line) with anti-MUC1 redirected T cells led to a viability rate of 24.9 ± 9.19%, while T47D cell viability was 85.01 ± 3.18% upon co-culture with untransduced T cells. These results were significantly higher than the viability of T47D cells co-cultured with MUC1- specific CAR-T cells (P<0.01). A negative control of a single culture of T47D cells showed 92.39 ± 3.76% viability (Fig.6A). In addition, as shown in Figure 6B, MCF-7 cells (another MUC1-positive tumour cell line) co-cultured with MUC1-specific CAR-T cells showed significantly lower viability (22.73 ± 5.24%) than the MCF-7 cells co-cultured with untransduced T cells (86.9 ± 17.81%, P<0.05) or MCF-7 single culture cells (92.31 ± 3.45%, P<0.01). There was no difference in the viability of A431 cells (a negative-MUC1 tumour cell line) while co-cultured with anti-MUC1 CAR-T cells (69.1 ± 22.48%) compared to the co-culture with untransduced T cells (70.93 ± 30.61%) or single culture cells (78.4 ± 17.55%), as seen in Figure 6C.

## Discussion

MUC1 is a heavily glycosylated glycoprotein with an extracellular domain that extends up to 200-500 nm from the cell surface. MUC1 has been found to be overexpressed on the cell surface in multiple epithelial aden(Sahraei, 2012 #64)ocarcinomas, including those of the breast, ovary, and pancreas (22). In malignant cells, MUC1 is re-distributed across the cell surface and loses apical-basal polarity that leads to interaction between MUC1 and few tyrosine kinase receptors such as epidermal growth factor receptor (EGFR) and platelet-derived growth factor-A (PDGFA), which then leads to cell proliferation and enhanced tumorigenesis. Furthermore, MUC1 promotes tumorigenesis through interactions with hypoxia-inducible factor a (HIF1-a) (23), increases angiogenesis (24) and inhibits the GSK3b pathway (25). In recent years, several clinical trials have used CAR technology for MUC1 targeting in solid and non-solid tumours (22).

CAR is composed of three main parts: i. Ligand binding domain that specifically recognizes tumour antigens and commonly is a B cell receptor (BCR)- derived scFv, ii. Extracellular spacer (hinge) which is the connecting region between the ligand binding domain to the transmembrane domain, and iii. Transmembrane and signalling domains. Between the hinge region and signalling endodomains are sequences which are usually derived from CD8, CD28 or CD3^+^ (transmembrane). Immediately after this region lies the signalling endodomains that transmit activation and costimulatory signals upon antigen recognition by scFv. According to these signalling endodomains, CARs are classified into three “generations”. Only CD3^+^ has been used as a signalling domain in “first generation” CARs. They lack co-stimulatory molecules and are unable to directing engineered T cell activation and expansion effectively upon antigen recognition. “Second generation” CARs are added to intracellular domain often comprising of CD28 or 4-1BB. "Third generation" CARs include CD3^+^ and two or more costimulatory domains, such as CD28 and usually OX40 (CD134) or 4-1BB (CD137) (26).

Although the use of third generation CARs has been more effective than second generation CARs based on their anti-tumour effects and persistence in animal studies (27), there were serious adverse events in a clinical trial conducted by Morgan et al. (28) (NCT00924287). They investigated efficacy and safety of third-generation anti- HER2 CAR and unfortunately their study terminated after a patient’s death due to CAR-T cell immunotherapy. The authors considered that the cause of the patient’s death was recognition of HER2-positive healthy lung epithelium by anti-HER2 CAR-T cells and respiratory failure. In contrast, significant toxicity has not been reported after infusion of second generation HER-2 specific CAR-T cells (NCT00902044) as the lack of 4-1BB in the endodomain of the CAR construct prevented excessive CAR-T cell activation (29). Therefore, it seems second generation CARs are a more suitable choice due to higher safety in solid tumour CAR-T cell therapy. Here, we have utilized a second generation MUC1-specific CAR to reduce the possibility of chronic cytotoxicity due to excessive T cell activation.

Most clinical CAR-T cell studies have utilized murine-derived scFvs that lead to the elimination of the scFv-based CAR-expressing T cells by the host anti-mouse or anti-IgE antibodies (30). These anti-CAR immune responses represent a challenge for the CAR-T cell-based treatments because longterm persistent engineered T cells are essential for the effective tumour elimination. A novel approach to reduce anti-CAR responses is VHH-based CARs. VHHs have high amino acid similarity to human VH family III. Therefore, in comparison with murine scFvs, humanization of these novel targeting antibodies is simpler and more efficient. Regarding the structure of VHH, it can be predicted that most substitutions with human sequence, except for the common key amino acids in the framework region 2 (FR2), can be implemented without altering its function and properties. Therefore, high homology with human antibody along with other features like small size (2.5 nm diameter and about 4 nm height), steric monomeric behaviour and high solubility, make VHHs ideal choices for cancer immunotherapy (31, 32). Hence, in this study, we have used a novel VHHbased anti-MUC1 CAR construct and subsequently transduced human primary T cells by recombinant lentiviruses that contained this gene construct.

To improve the condition of the high titer virus production, first we optimized the condition of transfection for our third generation of lentiviral vectors. Our data showed maximum transfection rate along with minimum toxicity at the 1:1 (PEI:DNA) ratio and 6 hour incubation period. Although reagent cytotoxicity increased with increasing PEI:DNA ratio levels, GFP expression did not increase in ratios higher than 1:1 PEI:DNA. Xie et al. (33) have reported that higher PEI concentrations are needed for an efficient transfection and suggested a 5:1 PEI:DNA ratio for optimum transfection. Differences in cell line type and experimental scale might be responsible for the different results. Next, we calculated recombinant lentivirus copy number by quantitative real-time PCR. The conventional method for determining the viral titer is the evaluation of GFP expression, but since the EGFP gene was removed from the pLJM1-EGFP after subcloning the CAR construct, we calculated the titer of the recombinant lentiviruses by evaluating puromycin resistant gene quantitation. Our results indicated that our novel VHH-based anti-MUC1 CAR-T cells could produce cytokines and cytotoxic activity, which are essential for a prosperous T cell based therapy. Successful clinical usage of CAR-T cells depends on high-level expression of CAR on the T cell surface. Our data showed 85% CAR expression on the T cells. Low expression of CAR is among the many challenges of CAR-T cell therapy and is a major obstacle. In several studies that used non-viral methods, the reported CAR expression was <50% (34, 35). Here, we used a third generation lentiviral vector with high MOI to transfer our *CAR* gene construct to human primary T cells. Moreover, we also optimized our T cell transduction method (data not shown). This high anti-MUC1 CAR expression was responsible for specific cytotoxicity and cytokine production observed upon co-cultivation of anti- MUC1 CAR expressing T cells with cancerous cells. Moreover, our results showed significantly reduced survival of MUC1-positive cells after co-culturing with transduced T cells (24.9% and 22.73%), but not in co-culturing with untransduced T cells (85.01% and 86.9%), which showed that specific recognition of the MUC1 antigen by anti-MUC1 CAR on the transduced T cells triggered intracellular signal transduction and activation of redirected human T cells, and led to specific elimination of MUC1-positive cells by these anti-MUC1 CAR-T cells. Accordingly, recognition of MUC1 by our anti-MUC1 VHH antibody was in an antigen-specific manner. In line with this result, we previously demonstrated specific and efficient recognition of a tumor antigen (TAG-72) by CCRFCEM cells that expressed another VHH-based CAR (anti-TAG-72), which led to proliferation and cytokine release (16).

In several CAR constructs, tonic signalling has led to prolonged expansion, constitutive cytokine release and exhaustion of T cells in the inexistence of target ligand and can be a cause of poor antitumor efficacy (36-38). Ligand-independent tonic signalling may occur due to clustering of CAR surface molecules, owing to the level of CAR surface expression as well as oligomerization and aggregation of the utilized scFvs (18). Structural properties of scFvs such as unfolded VH:VL flexible linker can cause domain swapping between the adjacent scFv molecules and lead to oligomerization (39). Several strategies have been suggested to improve scFv stability and thus prevent the scFvs oligomerization. These strategies include engineering of disulphide bonds in the linker between the VH and VL domains, computational modelling and selection of new target epitopes in antigens. It has been reported that substitution of the targeting domain with a single-chain antibody such as VHHs in the CAR structure may also avoid tonic signalling (18). VHHs are intrinsically incapable of domain swapping and oligomerization. Moreover, their small size enables VHHs to access the cryptic epitopes or large structures (40). It has been shown that the use of an SIN lentiviral vector for CAR expression may reduce tonic signalling and improve antitumor efficacy (36). According to our VHH-based anti-MUC1 CAR construct and the use of an SIN lentiviral vector, our functional assay results showed that, besides the high level of CAR expression, tonic signalling did not occur in the absence of the MUC1 antigen. It has been reported that the incorporation of a CD28 costimulatory molecule as the endodomain signalling resulted in tonic signalling of CAR-T cells. Long et al. (37) indicated that the replacement of the CD28 molecule with 4-1BB in a second generation CAR construct reduced T cell exhaustion. This contradicted our findings since our CD28-based anti-MUC1 CAR construct did not show the background cytokine production in co-incubation with MUC1-negative cells. In line with our results, Zhong et al. (27) did not observe any difference between CD28-CAR and 4-1BB CAR in the efficacy of tumour eradication.

## Conclusion

Taken together, the present study successfully demonstrated transduction of a second generation VHHbased CAR construct into human primary T cells. VHHbased anti-MUC1 redirected T cells were activated upon recognition of MUC1 expressing cancer cells and showed significant cytokine production and cytotoxic activity. This study supported the idea that VHH-based CARs might be a promising alternative strategy to efficiently target tumour cells and potentially overcome immunogenic and target-independent signalling impediments observed in scFv-based CARs. Although this study was significant in targeting and cytolysis of MUC1 positive cell lines due to VHH-based CAR-T cells specific for TAA-MUC1, *in vivo* experiments must be performed to assess both efficacy and safety.
